# Uma Anomalia Coronária Rara no Adulto: Fístula Arteriovenosa de Grande Calibre

**DOI:** 10.36660/abc.20230307

**Published:** 2024-02-16

**Authors:** Pedro Garcia Brás, Duarte Cacela, Rui Cerejo, Rui Rodrigues

**Affiliations:** 1 Hospital de Santa Marta Departamento de Cardiologia Lisboa Portugal Hospital de Santa Marta – Departamento de Cardiologia , Lisboa – Portugal; 2 Hospital de Santa Marta Departamento de Cirurgia Cardíaca Lisboa Portugal Hospital de Santa Marta – Departamento de Cirurgia Cardíaca , Lisboa – Portugal

**Keywords:** Fístula Arteriovenosa, Doença das Coronárias, Ecocardiografia, Tomografia Computorizada Cardíaca, Angiografia Coronária

## Descrição do caso

Paciente do sexo masculino, caucasiano, 57 anos, com história pregressa de hipertensão e enfisema pulmonar, compareceu ao ambulatório de Cardiologia com dispneia aos esforços, edema periférico, ortopneia e fibrilação atrial de início recente, com sopro contínuo III/VI, levantando a possibilidade de comunicação arteriovenosa intratorácica.

**Figura 1:**
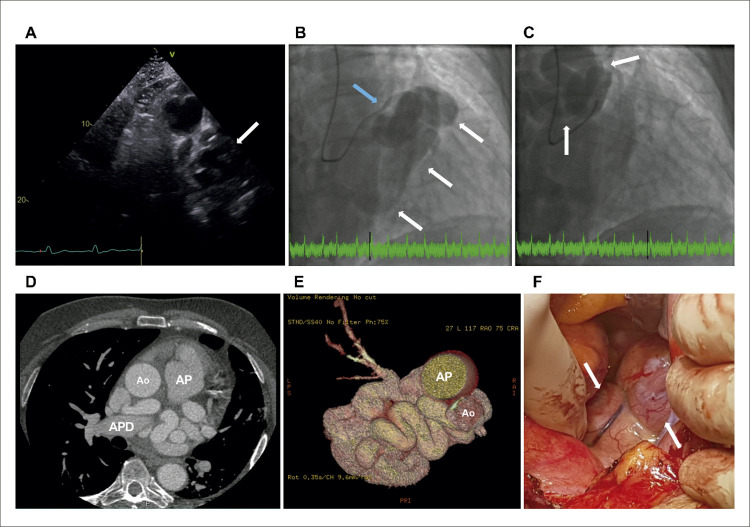
– A) Ecocardiografia transtorácica revelando múltiplas dobras tortuosas em formato de rosário (seta branca), levantando a possibilidade de fístula coronária. B) Preenchimento precoce com contraste na angiocoronariografia e C) preenchimento tardio com contraste mostrando fístula complexa de Sakakibara tipo B de grande calibre (setas brancas) originando-se do tronco da coronária esquerda (seta azul). D) Angiotomografia computadorizada cardíaca detalhando a anatomia tortuosa da fístula coronária entre o tronco da coronária esquerda e o seio coronário, com múltiplas pregas localizadas anteromedialmente à artéria pulmonar direita. E) Reconstrução tomográfica tridimensional da fístula coronária. F) Imagem intraoperatória do procedimento cirúrgico de obliteração da fístula mostrando os múltiplos trajetos tortuosos da fístula coronária (setas brancas). Ao: aorta; AP: tronco da artéria pulmonar; APD: artéria pulmonar direita.

Um ecocardiograma transtorácico revelou dilatação do ventrículo esquerdo com função sistólica global ligeiramente reduzida (fração de ejeção de 44%), dilatação biauricular e derrame pericárdico ligeiro. Notavelmente, este estudo mostrou múltiplas dobras ‘semelhantes a um rosário’ (Figura 1A, Vídeo 1 , Vídeos Suplementares S1-S2) com fluxo Doppler colorido, revelando uma possível fístula coronária. A relação Qp/Qs era normal. ^1 , 2^

**Vídeo 1:**
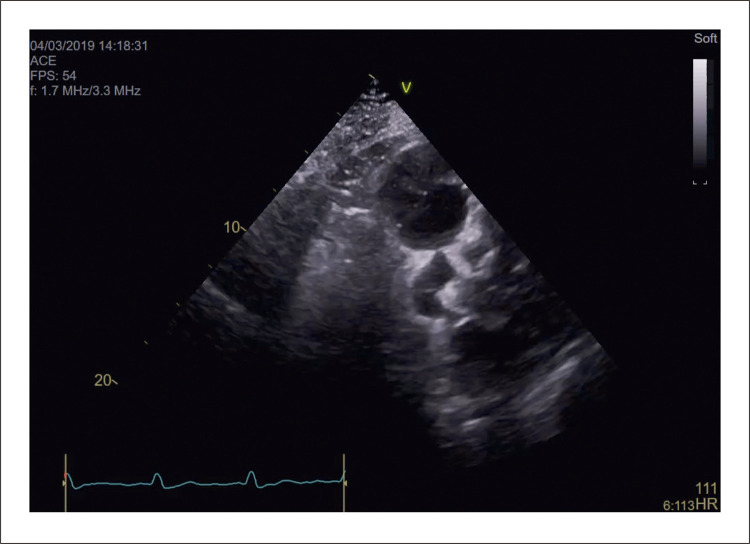
– Ecocardiograma transtorácico modificado em eixo curto basal (nível da válvula pulmonar) mostrando múltiplas dobras tortuosas em forma de rosário, sugestivas de fístula coronária. Link: http://abccardiol.org/supplementary-material/2023/12011/2023-0307_IM_Video_1.mp4

Também foi realizada cineangiocoronariografia (Figura 1B-1C, Vídeo 2 , Vídeo Suplementar S3), que confirmou a presença de uma fístula complexa de Sakakibara tipo B de grande calibre originada do tronco da coronária esquerda. ^1^ Uma TC cardíaca foi realizada para esclarecer a anatomia coronariana, mostrando uma fístula coronária tortuosa de grande calibre entre o tronco comum e o seio coronário composta por múltiplas pregas localizadas anteromedialmente à artéria pulmonar direita (Figura 1D-1E). O paciente iniciou terapia médica para insuficiência cardíaca e foi submetido com sucesso à obliteração cirúrgica da fístula coronária (Figura 1F, Vídeo 3 ). Em seguimento de 48 meses, o paciente encontrava-se em classe I da NYHA.

**Vídeo 2:**
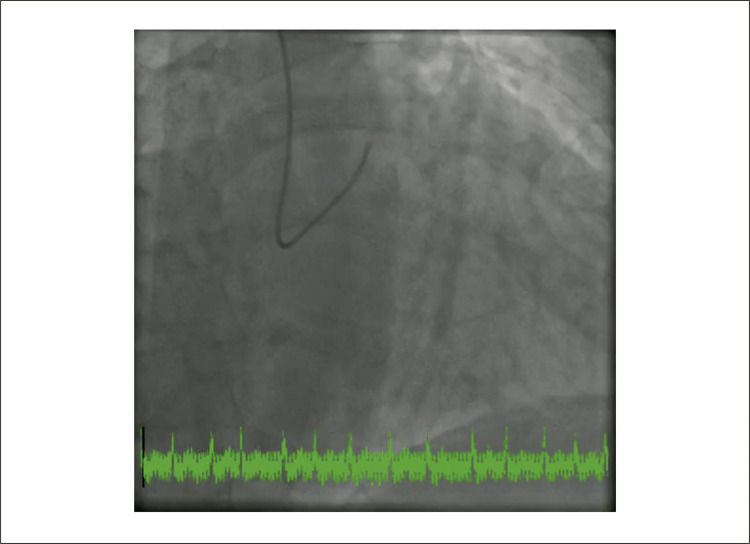
– Angiografia coronária. Injeção de contraste no tronco da coronária esquerda revelando a anatomia notavelmente tortuosa da fístula coronária originada do tronco da coronária esquerda. Link: http://abccardiol.org/supplementary-material/2023/12011/2023-0307_IM_Video_2.mp4

**Vídeo 3:**
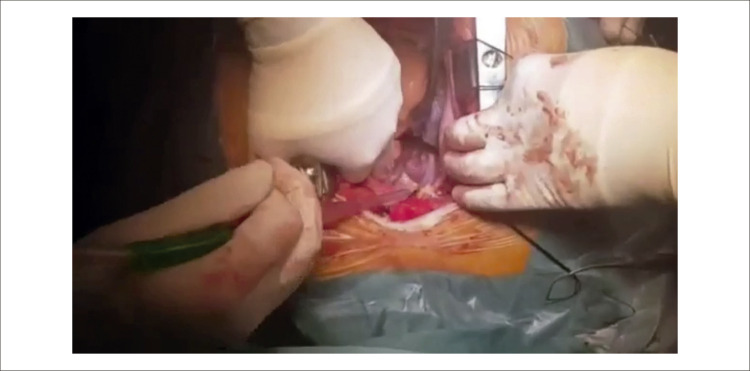
– Vídeo intraoperatório do procedimento cirúrgico de obliteração da fístula. Observe os múltiplos trajetos tortuosos da fístula coronária de grande calibre. Link: http://abccardiol.org/supplementary-material/2023/12011/2023-0307_IM_Video_3.mp4

As fístulas arteriovenosas coronárias são uma anomalia coronária rara, presente em 0,002% da população geral. ^3^ Embora a maioria das fístulas seja clinicamente silenciosa, os sintomas podem se desenvolver dependendo da extensão do shunt da esquerda para a direita ou da presença do fenômeno de roubo coronário, que geralmente se manifesta em idosos com insuficiência cardíaca congestiva, aterosclerose ou arritmias. ^2^
