# Supplementary material for: Uma Anomalia Coronária Rara no Adulto: Fístula Arteriovenosa de Grande Calibre
Source: Arq Bras Cardiol. 2024 Feb 16;121(1):e2023037. [Article in Portuguese] doi: 10.36660/abc.20230307 (PMC11098575; doi:10.36660/abc.20230307)
Supplement: Translation

# Translation: A Rare Coronary Anomaly in the Adult: Large-Caliber Arteriovenous Fistula

**Authors:** Pedro Garcia Brás, Duarte Cacela, Rui Cerejo, Rui Rodrigues

**Affiliations:** 1 Portugal Hospital de Santa Marta – Departamento de Cardiologia, Lisboa – Portugal; 2 Portugal Hospital de Santa Marta – Departamento de Cirurgia Cardíaca, Lisboa – Portugal

**Keywords:** Arteriovenous Fistula, Coronary Disease, echocardiography, Computerized Tomography Coronary Angiogram, Coronary Angiography

## Case description

A 57-year-old Caucasian male patient with a past medical history of hypertension and pulmonary emphysema presented to the Cardiology outpatient clinic with exertional dyspnea, peripheral edema, orthopnea, and new-onset atrial fibrillation, with a III/VI continuous murmur, raising the possibility of an intrathoracic arteriovenous communication.

A transthoracic echocardiogram revealed a dilated left ventricle with mildly reduced global systolic function (ejection fraction of 44%), biatrial enlargement, and mild pericardial effusion. Notably, this study showed multiple ‘rosary-like’ folds (Figure 1A, Video 2 , Supplementary Videos S1-S2) with color Doppler flow, revealing a possible coronary fistula. The Qp/Qs ratio was normal. ^1 , 2^

**Figure 1:** – A) Transthoracic echocardiography revealing multiple tortuous ‘rosary-like’ folds (white arrow), raising the possibility of a coronary fistula. B) Coronary angiography early contrast filling and C) late contrast filling showing a large-caliber complex Sakakibara type B fistula (white arrows) arising from the left main coronary artery (blue arrow). D) Multislice computed tomography angiography detailing the tortuous anatomy of the coronary fistula between the left main coronary artery and the coronary sinus, with multiple folds located anteromedially to the right pulmonary artery. E) Three-dimensional CT reconstruction of the coronary fistula. F) Intraoperative picture of the surgical fistula obliteration procedure showing the multiple tortuous pathways of the coronary fistula (white arrows). Ao: aorta; AP: pulmonary artery trunk; APD: right pulmonary artery.

**Video 2:** – Coronary angiography. Contrast injection in the left main coronary artery revealing the remarkably tortuous anatomy of the coronary fistula arising from the left main coronary artery. Link: http://abccardiol.org/supplementary-material/2023/12011/2023-0307_IM_Video_2.mp4

A coronary angiography was also performed (Figure 1B-1C, Video 3 , Supplementary Video S3), which confirmed the presence of a complex large-caliber Sakakibara type B fistula arising from the left main coronary artery. ^1^ A cardiac CT was performed to clarify the coronary anatomy, showing a large-caliber tortuous coronary fistula between the left main and the coronary sinus comprised of multiple folds located anteromedially to the right pulmonary artery (Figure 1D-1E). The patient was started on heart failure medical therapy and was successfully submitted to surgical ligation of the coronary fistula (Figure 1F, Video 3 ). In a 48-month follow-up, the patient was in NYHA class I.

**Video 3:** – Intraoperative video of the surgical fistula obliteration procedure. Note the multiple tortuous pathways of the large-caliber coronary fistula. Link : http://abccardiol.org/supplementary-material/2023/12011/2023-0307_IM_Video_3.mp4

Coronary arteriovenous fistulae are a rare coronary anomaly, present in 0.002% of the general population. ^3^ While most fistulae are clinically silent, symptoms may develop depending on the left-to-right shunt or the presence of coronary steal phenomenon, which usually manifests in older adults with congestive heart failure, atherosclerosis, or arrhythmias. ^2^
